# Differential toxicity profile of secreted and processed α-Klotho expression over mineral metabolism and bone microstructure

**DOI:** 10.1038/s41598-023-31117-6

**Published:** 2023-03-14

**Authors:** Joan Roig-Soriano, Cristina Sánchez-de-Diego, Jon Esandi-Jauregui, Sergi Verdés, Carmela R. Abraham, Assumpció Bosch, Francesc Ventura, Miguel Chillón

**Affiliations:** 1 Department of Biochemistry and Molecular Biology, Institut de Neurociènces (INc), Universitat Autònoma Barcelona, Bellaterra, Spain; 2 Departament de Ciències Fisiològiques, Facultat de Medicina i Ciències de la Salut, IDIBELL, Universitat de Barcelona, L’Hospitalet de Llobregat, Spain; 3 Departments of Biochemistry and Pharmacology & Experimental Therapeutics, Boston University School of Medicine, Boston, MA USA; 4 Vall d’Hebron Institut de Recerca (VHIR), Barcelona, Spain; 5 Centro de Investigación Biomédica en Red Sobre Enfermedades Neurodegenerativas (CIBERNED), Instituto de Salud Carlos III, Madrid, Spain; 6 Unitat Producció de Vectors (UPV), Universitat Autònoma Barcelona, Bellaterra, Spain; 7 Institució Catalana de Recerca i Estudis Avançats (ICREA), Barcelona, Spain

**Keywords:** Biochemistry, Drug discovery, Genetics, Molecular biology, Medical research, Molecular medicine

## Abstract

The aging-protective gene α-Klotho (KL) produces two main transcripts. The full-length mRNA generates a transmembrane protein that after proteolytic ectodomain shedding can be detected in serum as processed Klotho (p-KL), and a shorter transcript which codes for a putatively secreted protein (s-KL). Both isoforms exhibit potent pleiotropic beneficial properties, although previous reports showed negative side effects on mineral homeostasis after increasing p-KL concentration exogenously. Here, we expressed independently both isoforms using gene transfer vectors, to assess s-KL effects on mineral metabolism. While mice treated with p-KL presented altered expression of several kidney ion channels, as well as altered levels of P_i_ and Ca^2+^ in blood, s-KL treated mice had levels comparable to Null-treated control mice. Besides, bone gene expression of *Fgf23* showed a fourfold increase after p-KL treatment, effects not observed with the s-KL isoform. Similarly, bone microstructure parameters of p-KL-treated mice were significantly worse than in control animals, while this was not observed for s-KL, which showed an unexpected increase in trabecular thickness and cortical mineral density. As a conclusion, s-KL (but not p-KL) is a safe therapeutic strategy to exploit KL anti-aging protective effects, presenting no apparent negative effects over mineral metabolism and bone microstructure.

## Introduction

α-Klotho (KL) was discovered by Kuro-o et al. when studying a mouse strain that presented with a phenotype resembling accelerated human senescence^1^. This strain was characterized by a mutation in the regulatory region of the KL gene and generated a strong reduction of the Klotho protein production. Since then, several functions have been described for KL, being currently considered as a pleiotropic anti-aging hormone.

Klotho gene is mainly expressed in the kidney and in the brain choroid plexus, and it encodes for two transcripts^2^. The full length (m-KL) generates a type I transmembrane protein presenting two homologous extracellular domains, KL1 and KL2. The extracellular portion can be shed by proteolytic cleavage, generating processed KL (p-KL), also known as soluble KL, containing both KL1 + KL2 domains. Additionally, the KL1 and KL2 domains, can be shed generating independent soluble subunits^3^. Alternatively, a second transcript generates a shorter mRNA which presents a stop codon short after the KL1 domain, generating a secreted isoform (s-KL)^2^. Although recent reports doubt if this endogenous transcript produces a functional protein^4^, different groups have reported that increasing the levels of s-KL or KL1 through recombinant protein administration or gene transfer approaches confers clear beneficial properties^5–8^. Klotho can modulate several pathways related to aging progression, which include among others, the inhibition of insulin growth factor-1 (IGF-1)/insulin signaling and Wnt pathways, involved in senescence and fibrosis, respectively^9^. It also reduces oxidative stress and exhibits anti-inflammatory effects, reducing cellular damage during aging^10^. Besides, KL also plays a key role in mineral metabolism, where it is involved in direct modulation of ion channels, PTH hormone production, vitamin D regulation and fibroblast growth factor 23 (FGF23) hormone signaling^11^. For more details on KL anti-aging functions, see the review by Abraham and Li, 2022^12^.

Klotho has a direct impact over some ion transporter proteins increasing abundance and activity of these channels. Transient receptor potential cation channel subfamily V members 5 and 6 (TRPV5/6) calcium channels, potassium transporter renal outer medullary potassium channel 1 (ROMK1 or KCNJ1) and NaPi-2a co-transporter (SLC34A1), are activated and stabilized in the membrane by KL’s sialidase activity^13,14^. Moreover, KL is also involved in Na^+^, K^+^- ATPase channel transport to the membrane, responsible of Ca^2+^ reabsorption in the kidney^13^. Modulation of these channels represents a fast system of controlling ion concentrations.

In addition to this direct effect on ion transporters, KL also participates in metabolism of vitamin D (1,25D), which is responsible for increasing renal and intestinal absorption of Ca^2+^ and inorganic phosphate (P_i_)^15^. In turn, activation of vitamin D receptor (VDR) by 1,25D upregulates Klotho transcripts and FGF23 production^16^, which will inhibit further 1,25D synthesis, promoting a decrease of P_i_ levels^15^.

Finally, KL is also implicated in FGF23 production and signaling pathway. FGF23 is a bone-derived hormone important for phosphate concentration regulation^11^. After phosphate intake, FGF23 is secreted from osteocytes in the bones and reduces this ion concentration by different mechanisms. First, it increases renal phosphate excretion by downregulating NaPi-2a and NaPi2c (SLC34A3) ion channels. Secondly, it inhibits vitamin D activation, downregulating renal 1-alfa-hydroxylase (CYP27B1) and promoting vitamin D degradation by increasing 24-hydrolase (CYP24A1) enzyme^17^.

Klotho implication in FGF23 signaling was well known from the study of the phenotypes of the *klotho* knock out (KO) mice and the *Fgf23* KO mice. Both mutants presented hyperphosphatemia, increased vitamin 1,25D, ectopic calcifications, and extremely shortened lifespan^1,18,19^. In contrast, just Klotho overexpression increased lifespan, suggesting FGF23 is not related to this Klotho property^20,21^. In this system, transmembrane KL (m-KL) acts as an obligate co-receptor for FGF23 signaling. This is due to the interaction of Klotho with FGFR1, FGFR3 and FGFR4, receptor proteins responsible for FGF23 signaling. This interaction increases affinity of the FGF23 receptors for their FGF23 ligand, making possible the receptor activation. In a structural-molecular point of view, FGFR interacts with a region in KL2 domain, stabilizing the binding. Deletion of the receptor binding loop (RBL) region of KL2 inhibits Klotho-induced increase in phosphate urinary excretion, proving a decreased FGF23 activity^11^. Importantly, Klotho also affects FGF23 production, as exogenous administration of p-KL (or soluble KL) induced a significant increase in the FGF23 amount in serum, altering mineral metabolism^22^.

The pleiotropic beneficial properties of KL protein isoforms represent a promising strategy to treat different age-related diseases. However, due to the key role in mineral homeostasis, exploiting protective anti-aging effects of p-KL could in turn translate in deregulation of the bone ion regulation system. Currently, it is unknown whether the s-KL isoform also promotes FGF23 production in osteocytes in vivo and present the same toxic effect observed for p-KL. Here, we aimed to address this question.

## Results

### Gene therapy treatment efficiently overexpresses secreted and processed KL isoforms

**Figure 1:**
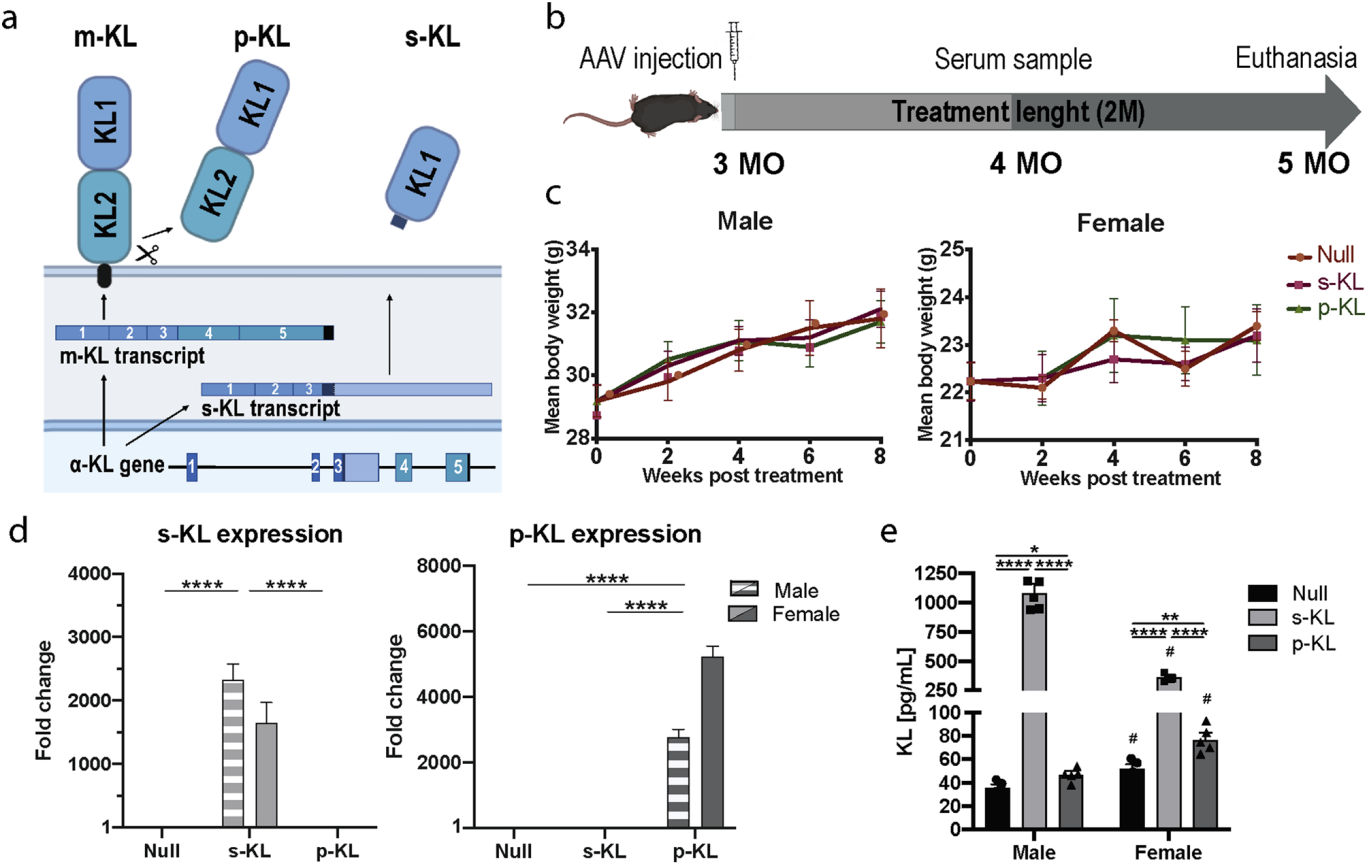
(**a**) Scheme with the different KL isoforms. (**b**) Experimental design followed. (**c**) Body weight follow up displayed as increase in grams during the 8 weeks-long treatment. No differences in body weight were observed between groups before the start of the experiment (t = 0) or after 8 weeks. (**d**) Gene expression of secreted and processed KL in liver 2 months after the treatment. Stripped bars represent gene expression in males and solid bars in females. Data presented as fold change expression compared to Null treated animals. (**e**) KL protein levels in serum 2 months after the treatment. Mean ± Standard error of the mean (SEM) for (**c,e**), and relative quantity (RQ) ± standard error of RQ for (**d**); n = 4–6; ^#^p < 0.05 for differences between males and females, *p < 0.05; **p < 0.01; ***p < 0.001; ****p < 0.001 for differences between different treatment groups within the same gender.

Expression cassettes under the control of the CMV promoter were cloned carrying Control-Null sequence (stuffer, non-coding sequence), secreted (s-KL), or processed KL (p-KL) sequences (Fig. 1a). Different adeno-associated viral vectors serotype 9 (AAV9) were generated for each construct and the animals were administered IV and ICV at the age of 3 months (Fig. 1b), without observed intervention-derived negative effects. Body weight increase at that age was not affected by the treatments as can be seen in the follow-up done during the 2 months-long treatment (Fig. 1c). As expected after an intravenous injection, AAV9 vectors efficiently increased viral vector expression in liver, similarly in male and female mice (Fig. 1d), which in turn led to increased levels of KL isoforms in serum (Fig. 1e). Both isoforms were increased compared to Null group levels, although secreted isoform displayed higher levels both in males and females, compared to the processed KL isoform. Of note, females presented higher basal KL levels compared to males treated with Null vector.

### The secreted KL isoform does not alter ion metabolism in young C57BL/6J mice

**Figure 2:**
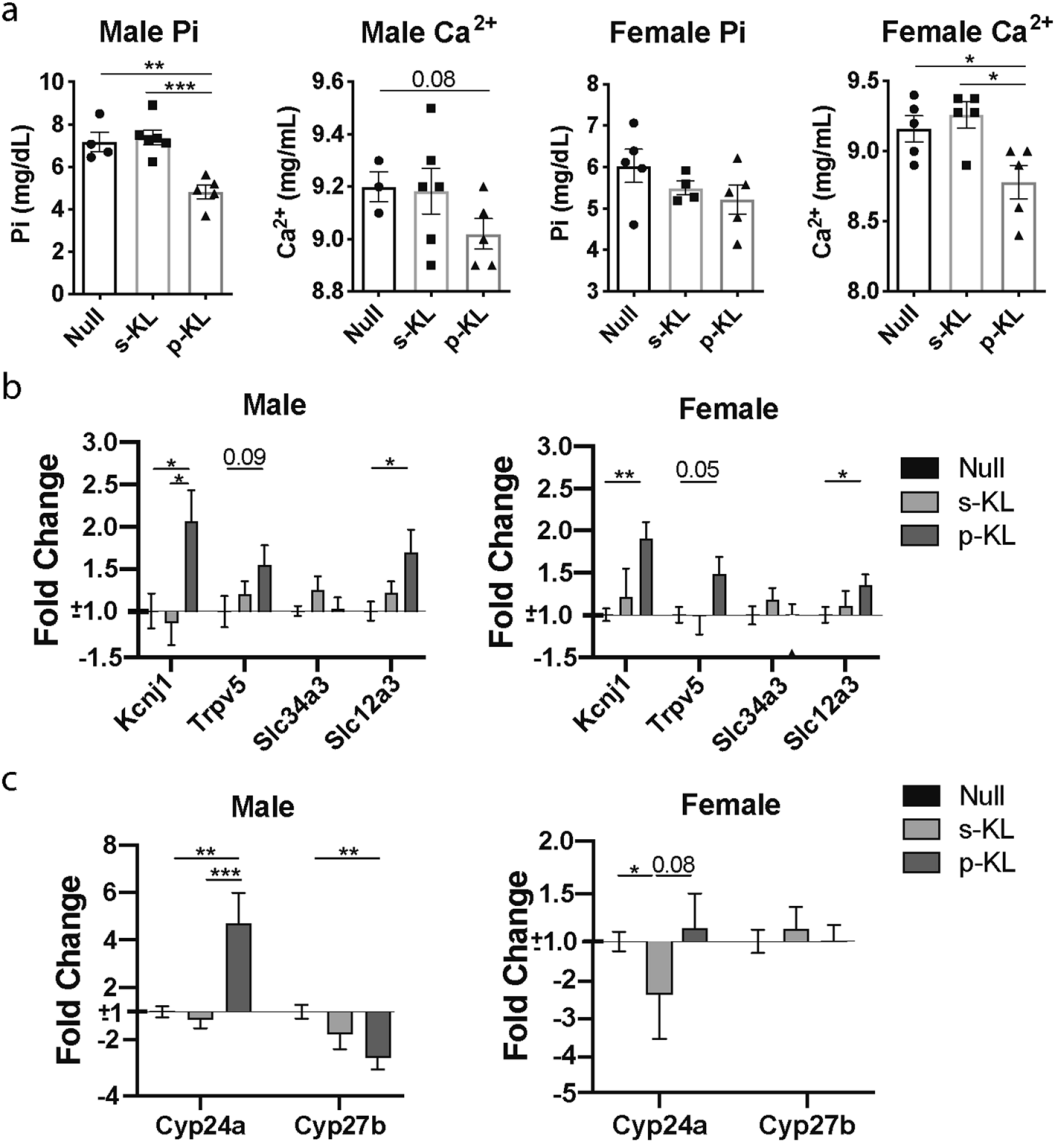
(**a**) Ca^2+^ and Pi serum levels after 8 weeks of treatment. (**b**) Expression of ion channels in kidney. (**c**) Expression of genes involved in vitamin 1,25D activation and degradation. Data are presented as fold change expression compared to Null-treated animals. Mean ± Standard error of the mean (SEM) for (**a**), and relative quantity (RQ) ± standard error of RQ for (**b,c**); n = 4–6; *p < 0.05; **p < 0.01; ***p < 0.001 for differences between the treatment groups.

Serum of treated animals was analyzed to study alterations in ion homeostasis. Eight weeks after vector administration, p-KL treated animals showed a decrease in phosphate (P_i_) and calcium (Ca^2+^) ions concentration, reaching significant differences compared to control and s-KL treated animals (Fig. 2a). Also, as seen in Fig. 2b, only p-KL altered the expression of different ion channels. ROMK and sodium chloride cotransporter SLC12A3 (also known as NCC) were significantly increased, and the TRPV5 channel exhibited a trend both in males and females. In contrast, no changes were observed after s-KL treatment. Of note, no expression changes were observed for the *Fgfr* genes (data not shown).

Enzymes of Vitamin D metabolic pathway were also affected after treatment. In kidney, 25(OH)D, precursor for vitamin D, is either enzymatically activated (CYP27B) or inactivated (CYP24A). Interestingly, after AAV administration, males treated with p-KL presented an increase in the expression of the inactivating *Cyp24b* gene, while the vitamin D synthesis gene *Cyp27b*, was reduced. In contrast, females treated with s-KL presented a significant reduction in the expression of the vitamin D catalytic *Cyp24a* gene, compared to Null and p-KL animals. No changes were observed for *Cyp27b* gene in s-KL treated animals (Fig. 2c).

### Effect of KL-expressing vectors in bones

**Figure 3:**
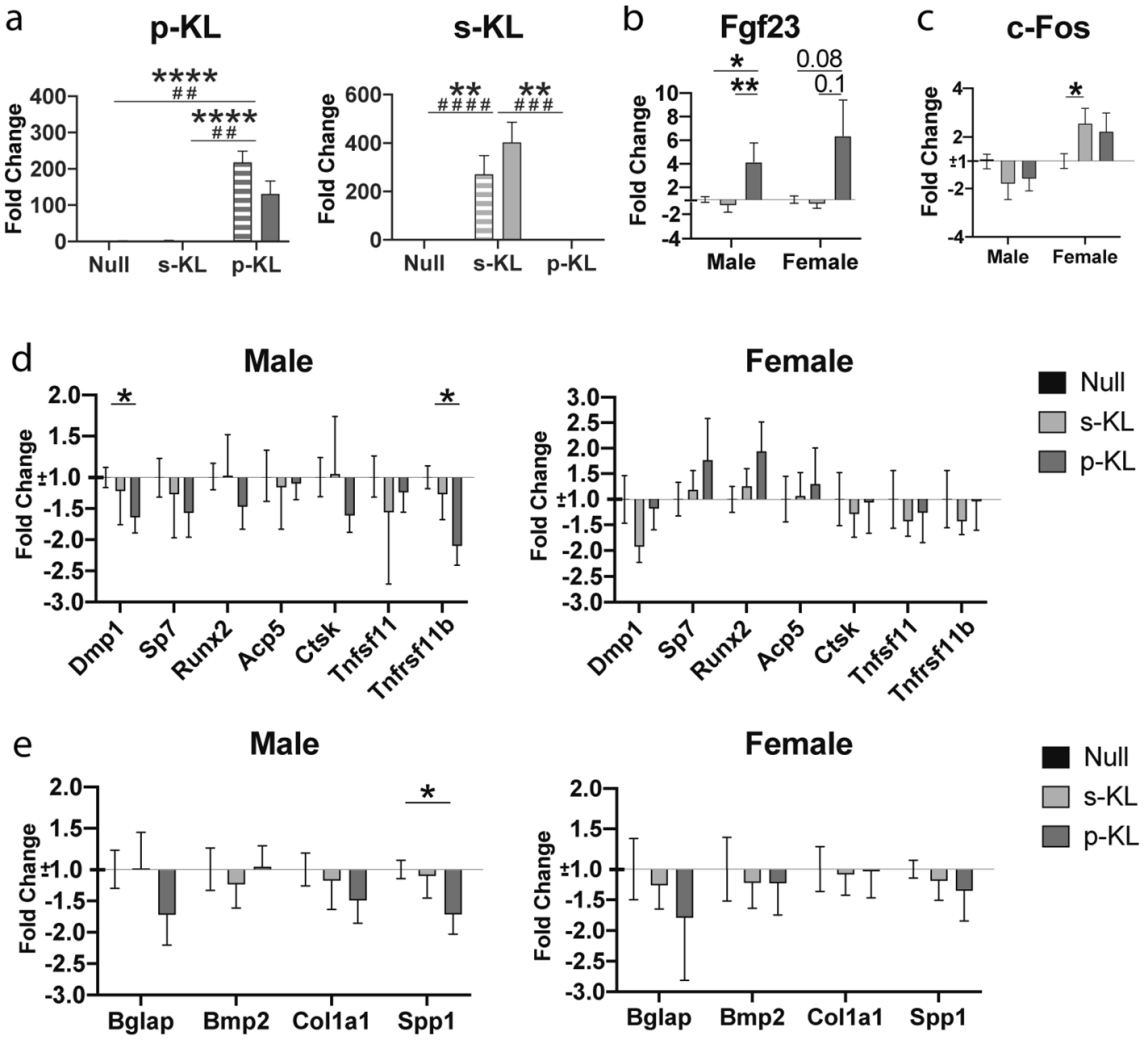
(**a**) Gene expression of secreted and processed KL in bone tissue 2 months after the treatment. Stripped bar represents gene expression in males and solid bar in females. Statistical significance presented as (*) for representing differences between treatment in male groups, and (#) for comparing treatments in females. (**b**) Effect of treatment on *Fgf23* gene expression. (**c**) Expression analysis of Klotho down-stream gene *c-Fos*. (**d**) Effect of treatment on cell type specific gene expression. (**e**) Effect of treatment on bone protein matrix gene expression. Data are presented as fold change expression compared to Null treated animals. Relative quantity (RQ) ± standard error of RQ; n = 4–6; *p < 0.05; **p < 0.01; ***p < 0.001; ****p < 0.001 for differences between the treatment groups.

Intravenous administration of AAV9 viral vectors also allowed transduction of the bone tissue, increasing more than 100-fold over basal expression of Klotho isoforms in both sexes. No significant differences were observed in the overexpression between genders (Fig. 3a). *Fgf23* gene was strongly affected by the treatment. p-KL generated a four and six-fold increase in males and females, respectively. Interestingly, the secreted isoform s-KL did not alter *Fgf23* expression (Fig. 3b). In turn, both KL isoforms activated typical KL downstream signaling, with a tendency to decrease *c-Fos* expression in males, and increasing its expression in females, reaching significance for s-KL isoform (Fig. 3c).

The expression of different genes related to lineages of osteocytes (*Dmp1*), osteoblasts (*Sp7* and *Runx2*), osteoclasts (*Acp5* and *Ctsk*) and genes related to bone resorption *Tnfsf11* (*RankL*) and *Tnfrsf11b* (*Opg*) were not statistically modified by the treatment (Fig. 3d), except for a significant reduction of *Dmp1* and the bone resorption protective protein OPG, in p-KL treated males. Expression of osteocalcin (*Bglap*), *Bmp2*, *Col1a1* and osteopontin (*Spp1*) genes, involved in bone structure was also assessed (Fig. 3e). A significant reduction in Spp1 and a clear tendency to a reduced *Bglap* expression were observed in p-KL treated males, while no effects were observed in s-KL treated animals.

### Klotho treatments affect differently bone structure and physical properties

**Figure 4:**
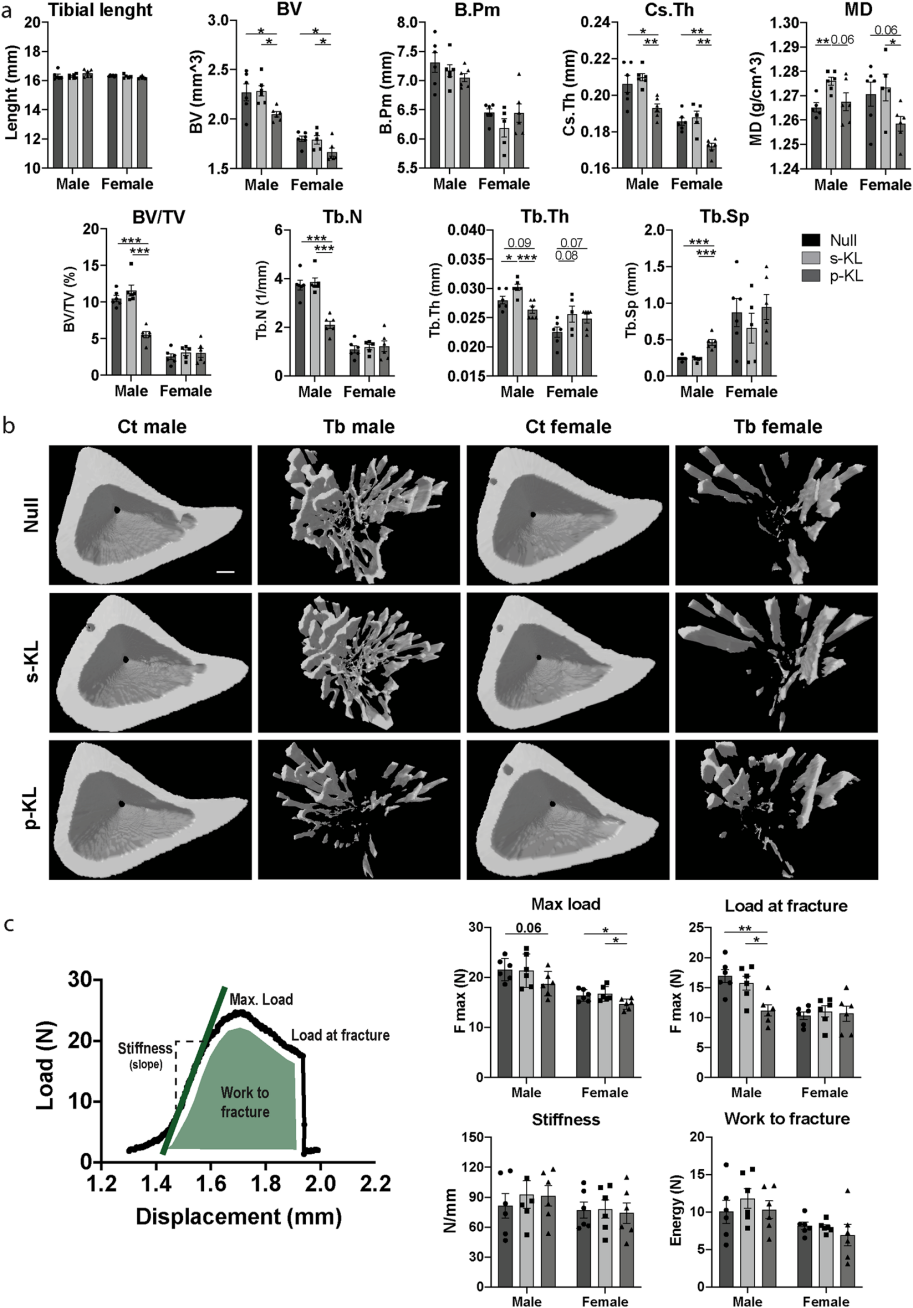
(**a**) MicroCT analysis of different structural variables. Tibial length, BV (bone volume), B.Pm (bone perimeter), CsTh (cross-sectional thickness), Mineral Density (MD), BV/TV (bone volume/tissue volume), Tb.N (trabecular number), Tb.Th (trabecular thickness), Tb.Sp (trabecular space). (**b**) 3D reconstruction of the analyzed ROI. First and third columns corresponding to cortical bone and second and fourth to trabecular bone. Scale bar = 0.2 mm. (**c**) Results of three-point bending test. Data represented as maximum load resisted by the bones, force at fracture, stiffness, and total energy absorbed by the bones during the experiment. Data are presented as fold change expression versus Null treated animals. Mean ± Standard error of the mean (SEM), n = 4–6; *p < 0.05; **p < 0.01; ***p < 0.001; ****p < 0.001 for differences between the treatment groups.

The effects of overexpression of the different Klotho isoforms on bone microstructure was assessed by MicroCT analysis. Specifically, cortical bone, medullar cavity and trabecular bone parameters were measured. As observed in Fig. 4a, the p-KL isoform had a significant impact over cortical bone, reducing bone volume and cortical thickness by more than 10%, both in males and females. Interestingly, treatment with s-KL increased cortical mineral density (MD) compared to Null-treated mice. No changes were detected in bone length, perimeter or endocortical volume. As expected, males exhibited significantly higher levels of trabecular volume than females, even in the Control-Null group. In accordance with cortical bone, p-KL treatment in males also reduced trabecular bone volume (BV/TV), trabecular number and thickness, and increased trabecular spacing. Interestingly, the s-KL isoform did not induce this osteopenic phenotype and, in fact, even significantly increased the trabecular thickness compared to control mice (Fig. 4a). These structural differences can be seen in the 3D reconstruction of the ROI analyzed (Fig. 4b).

Finally, a three-point bending test was done in order to assess the effect of these structural changes on bone physico-mechanical properties (Fig. 4c). Results showed that the p-KL treatment reduced the maximum load the bones could resist before starting to bend. Moreover, a reduction in load at fracture was also observed in males. In contrast, the s-KL treatment did not reduce neither maximum load, nor load at fracture, thus demonstrating a safer profile than the p-KL treatment. Lastly, s-KL and p-KL did not induce significant changes to stiffness, elasticity (displacement pre-fracture) and total energy absorption.

## Discussion

Klotho isoforms are known as potent pleiotropic anti-aging proteins, which could be used as a treatment for different age-related conditions. However, its implication in mineral homeostasis requires special attention to avoid undesired deregulation of the ion balance.

After the eight weeks long treatment, AAV vectors efficiently expressed KL isoforms in the different tissues analyzed that translated into increased basal KL protein levels in serum of treated animals. Although p-KL gene expression in liver resulted in higher levels, s-KL isoform treatment generated significantly more protein in serum. The secreted isoform is shorter than processed KL, which could increase the translation and secretion rate of the protein, and explain the higher levels observed. Moreover, p-KL can interact with FGFR and could be partially retained in cell membranes. However, even though it reached lower protein levels, p-KL protein had a large impact in calcium (Ca^2+^) and phosphate (P_i_) homeostasis, significantly decreasing their levels in serum. These results are in agreement with Smith et al., which described altered ion balance after p-KL expression^22^. In contrast, s-KL did not significantly alter ion levels, proving that the KL2 domain, found only in p-KL, is needed for this effect. To our knowledge, this is the first time this effect is described and compared for the s-KL and p-KL isoforms.

Activation of the FGFR/KL complex by FGF23 decreases phosphate levels by first reducing Na-dependent P channels and, secondly, by inhibiting vitamin D metabolism^14^. In this study, expression of *Slc34a3* channel (also known as *Npt2c*) did not change after upregulation of both KL isoforms. In contrast, KCNJ1 (ROMK channel), in charge of secreting potassium into the urine, was upregulated in p-KL-treated animals, which is in agreement with Cha and collaborators, who reported that KL administration increased KCNJ1 channels in distal nephrons in the kidney, increasing potassium secretion to urine^23^. TRPV5 channel, which reabsorbs calcium in the kidney, also tended to increase in p-KL treated males and females. This effect could be a compensatory mechanism to recover decreased Ca^2+^ levels in p-KL animals. Additionally, NCC channel (SLC12A3), which participates in reabsorbing sodium and chloride ions from urine, was also upregulated. This was also observed in the KL-overexpressing transgenic mouse^24^. Of note, ion channel alterations were not observed after the s-KL treatment suggesting no negative effects of this isoform on the kidney transporters studied here.

Secondly, enzymes related to vitamin 1,25D activation, responsible for increasing calcium and phosphate accumulation, were also studied in kidney. Vitamin D activator CYP27B and degrading CYP24A enzymes were significantly down and upregulated, respectively, after p-KL treatment in males. This result agrees with the observed reduced phosphate and calcium levels in blood. Again, s-KL had no effect over 1,25D metabolism. Interestingly, s-KL treatment in females decreased vitamin D degrading CYP24A enzyme, although this did not translate into higher ion levels compared to Null mice. More specific research is needed to understand the different effects of p-KL and s-KL isoforms that are gender-specific.

Based on the results obtained, we hypothesize that these alterations observed in FGF23 signaling are due merely to the increased FGF23 levels in p-KL treated mice, and not due to increased KL-dependent activity of the FGF23 receptor. Previous reports already provided evidence that p-KL increased FGF23 production, specifically by osteoblasts in bones. This generated hypophosphatemia and low 1,25D levels, provoking osteoporosis and osteomalacia^22^. This effect was independent of p-KL effect over KL/FGF23 signaling because, although p-KL can bind to FGFR, it is thought to not transduce intracellularly the FGF23 signal^24^. This finding prompted us to try to address this effect with additional KL isoforms and subunits. To test whether the s-KL isoform affected or not *Fgf23* expression, we analyzed the expression of key genes in bones. In our model, p-KL overexpression increased 4 times the *Fgf23* expression compared to control-Null mice. This result agrees with Smith et al., who described a FGF23 upregulation after a p-KL (soluble KL1 and KL2 domains) treatment^22^. Importantly, s-KL did not cause this increase in *Fgf23* expression, which could explain why s-KL does not produce a negative effect on mineral homeostasis. In turn, both isoforms were able to affect *c-Fos* expression, the downstream KL pathway, confirming both KL isoforms are functional, although only p-KL induces FGF23 upregulation. Klotho-mediated upregulation of *Fgf23* expression, was also observed in the KL-overexpressing mouse model, which also exhibits elevated FGF23 levels. Contrary, this mouse model did not present bone problems or phosphate dysregulation. This could be due to compensatory mechanisms present in the transgenic strain, like the observed reduction in 1,25D and parathyroid hormones and the regulation of phosphate channels expression^25^.

We also tested the possible side effects of KL treatment over bone microstructure, by doing a microCT analysis of the long bone tibia. As previously described, p-KL treatment had a big osteopenic impact over bone mineral density and microstructure, in both cortical and trabecular bone^22^. Both genders presented similar effects, although females at a lower magnitude, which could be related to the lower trabecular bone mass, characteristic of female bones.

Interestingly, the s-KL treatment not only lacked negative side effects, but exhibited an improvement in bone status increasing bone mineral density and trabecular thickness in male mice. Previous reports from our group already described a possible positive effect of s-KL isoform over-expression in bones. Long bones of aged SAMP8 mice treated with s-KL AAVs presented microstructure values comparable to the healthy control SAMR1, while Null-vector treated mice presented values equivalent to aged SAMP8 mice^5^.

In addition, in p-KL treated males the effects over bone microstructure were accompanied by a reduction in expression of genes characteristic of osteocytes and bone resorption, and further accompanied by a decreased expression of osteopontin (*Spp1*) and osteocalcin genes (*Bglap*), both components of the bone structure. This finding together with the reduced ion availability could explain the observed alterations in the bones of p-KL-treated animals. Microstructure alterations caused by p-KL provoked a negative effect over bone tissue function. As observed, the p-KL treatment led to weaker bones, exhibiting decreased supported maximum load and load at fracture values, confirming specific negative side effects associated with high levels of this isoform.

Different previous studies had reported negative effects of exogenous KL administration over bone mineralization, discouraging its use as an anti-aging treatment^26^. This effect was also described for humans, since a mutation leading to a strong increase of p-KL in serum also induced hypophosphatemia and hyperparathyroidism^27^. On the other hand, several studies emphasize the importance and beneficial effects of Klotho for correct bone homeostasis, as knockout (KO) Klotho mice show important osteoporotic phenotype attributed to ion deregulation^1^. Moreover, Klotho is involved in the correct expression of non-collagenous bone proteins and bone microstructure formation. As such, KO Klotho mice present altered BGLAP and DMP1 protein microstructure^28^. Interestingly, the s-KL isoform has been reported to increase expression of these structural genes in in vitro osteoblast cultures^29^. Although that effect was not reproduced in our in vivo experiment (Fig. 3e), we have detected improved trabecular and mineral density in bones of male mice treated with s-KL. RNA expression analysis of just osteoblasts could provide with more precise information to elucidate the mechanisms of improvement in those variables.

In summary, in this work, we have shown evidence that high s-KL levels do not induce the deleterious side effects of p-KL over ion balance and bone homeostasis, while high p-KL levels lead to a decrease in calcium and phosphate ions and alter expression of 25(OH)D, renal ion-transporters and channels. These changes led to reduced bone tissue and weaker bones. In contrast, s-KL did not alter those pathways and even improved some of the bone variables, although more research is needed to better understand the effect of s-KL over bone tissue microstructure. In conclusion, high levels of s-KL, but not p-KL, present a good biosafety profile, which facilitates its use as a long-term therapeutical molecule to treat aging-associated deficits.

## Material and methods

### Animal housing

C57BL/6J male (n = 18) and female mice (n = 18) were purchased from Charles River. These animals were randomly divided into 3 groups: Null control mice (n = 6), s-KL (n = 6) or p-KL (n = 6) treated animals per sex. Mice had free access to food and water and were kept under standard temperature conditions (22 ± 2 °C) and a 12-h light/dark cycle (300 lx/0 lx). Gene therapy approach consisted in expression cassettes under the control of the CMV promoter containing secreted (s-KL) or processed (p-KL) isoforms of the mouse α-KL gene or a control-null sequence. Three adeno-associated viral vectors serotype 9 (AAV9), containing independently those constructs, were generated at the Unitat de Producció de Vectors (UPV) (www.viralvector.eu) following the triple transfection method^30^. Animals were administered simultaneous by intracerebroventricular (ICV) and intravenous (IV) injection, to transduce as many tissues as possible. Intracerebroventricular stereotaxic injections of AAV vectors were performed as previously described^5^. Briefly**,** the treatment was administered into the right hemisphere at coordinates, − 0.2 mm antero-posterior, − 2 mm dorso-ventral, and + 1 mm medio-lateral from bregma. The vector dose was 1 × 10^11^ viral genomes per animal in 6 μl, administered at a 0.5 μl/min speed using an ultramicropump (WorldPrecision Instruments). The intravenous injection consisted in a dose of 4 × 10^11^ viral genomes per animal diluted with NaCl 0.9% to a final volume of 200 μl, and injected manually with a syringe into the lateral tail vein of the mice. After the two months long treatment, animals were deeply anaesthetized with 4–5% Isoflurane and sacrificed by decapitation, according to ethical procedures.

### Serum biochemical analysis

Blood samples were obtained by decapitation, with a Serum Separation Tube (BD microtainer). Blood was left at room temperature for 5 min and then placed on ice. Blood serum was isolated by centrifugation at 3000 rpm for 15 min, and finally aliquoted and kept frozen at − 80 °C. Calcium and phosphate serum levels were measured at the Servei de Bioquímica Clínica Veterinària at the UAB, at 340 nm following the Arsenazo III and Phosphomolybdate methods, respectively. KL serum levels were measured using an ELISA kit specific for mouse KL (IBL) following manufacturer’s indications.

### Gene expression

Total RNA isolation was carried out using TRIsure™ reagent following the manufacturer’s instructions (Bioline Reagent). The tissues used for the extraction were liver, half kidney, and long bones (tibia and femur without medulla). Tissues were homogenized using TissueLyser LT sample disruption apparatus (QIAGEN). RNA quantity and purity were measured with NanoDrop™ 1000 Spectrophotometer (Thermo Scientific). RNA retrotranscription was done using iScript™ Advanced cDNA Synthesis Kit (Bio-rad). Gene expression was analyzed by Real-Time quantitative PCR (RT-qPCR) on a Bio-Rad CFX-384 PCR machine at the Analysis and Photodocumentation Service of the Universitat Autonòma de Barcelona. Each reaction contained 25 ng of cDNA, 7.5 μl of iTaqTM Universal SYBR Green Supermix (Bio-Rad) and a primer concentration of 0.2 nM, with a final reaction volume of 15 μl. In case of TaqMan probes (ThermoFisher scientific), 5 μl of master mix (Norox), 0.5 μl TaqMan mix and H2O to a final volume of 10 μl were used. Primers and TaqMan used are listed in Tables 1 and 2. The actin b (Actb) gene was used as a housekeeping control for qPCR done with SYBR green. The TATA binding protein (Tbp) gene was used as a housekeeping control for qPCR using Taqman done with RNA from bones, and Gapdh gene for Taqman qPCR done with RNA from the kidneys and liver. The analysis of the results of qPCR was done following the ΔΔCt method. Briefly, the cycle threshold (Cq) value of each gene of interest was normalized by subtracting the difference of the housekeeping gene (HK) Cq of each sample with respect the HK’s average value. Finally, data were presented as fold change in expression compared to Null-treated animals.

**Table 1:** Sequence of SYBR green primers used.

Target	Forward primer	Reverse primer
Cyp24a1	CTGCCCCATTGACAAAAGGC	CTCACCGTCGGTCATCAGC
Cyp27b1	GCACAGTTTACGTTGCCGAC	CGTTAGCAATCCGCAAGCA
s-KL	TCATAATGGAAACCTTAAAAGCAA	CACTGGGTTTTGTCAAAGGA
p-KL	TACGGAGACCTCCCGATGTA	CGCAAAGTAGCCACAAAGGT
Actb	CAACGAGCGGTTCCGAT	GCCACAGGTTCCATACCCA

**Table 2:** References of Taqman probes used.

Target	Taqman reference
*Acp5*	Mm00475698_m1
*Bglap*	Mm03413826_mH
*Bmp2*	Mm01340178_m1
*c-Fos*	Mm00487425_m1
*Col1a1*	Mm00801666_g1
*Ctsk*	Mm00484039_m1
*Dmp1*	Mm01208363_m1
*Egr1*	Mm00656724_m1
*Fgf23*	Mm00445621_m1
*Gapdh*	Mm 99999915_g1
*Slc12a3 (NCC)*	Mm01173990_m1
*Slc34a3 (Npt2c)*	Mm00551746_m1
*Runx2*	Mm03003491_m1
*Kcnj1 (Romk)*	Mm01173990_m1
*Spp1* (Opn1)	Mm00436767_m1
*Sp7* (Osx)	Mm00504574_m1
*Tbp*	Mm00446973_m1
*Tnfsf11* (RANKL)	Mm00441906_m1
*Tnfrsf11b* (Opg)	Mm00435454_m1
*Trpv5*	Mm01166037_m1

### MicroCT

Bone microstructure study was done as previously described^5^. Briefly, right legs of perfused animals were isolated, fixed with PFA 4% solution and stored in PBS solution with 0.05% sodium azide (NaN3). MicroCT analysis was done with a SkyScan 1272 (Bruker) computerized microtomography imaging system at the Centre de Recerca en Ciència i Enginyeria Multiescala de Barcelona (CRCEMB) at Universitat Politècnica de Catalunya (UPC). Images were reconstructed with the NRecon v1.6 (Bruker) program and analyzed with the CT-Analyser v1.13 image program (Bruker). Finally, 3D representations of the bones were obtained with the CTVox v3.3 program (Bruker). Mineral density was calculated with the CT-Analyzer v1.13 program calibrating bone absorbance with two 2 mm diameter hydroxyapatite phantoms (Bruker-MicroCT) of known density of 0.25 and 0.75 g/cm^3^.

### Three-point bending test

Bone resistance to fracture was measured by three-point bending test done at the Biomaterials, Biomechanics and Tissue Engineering Group, Technical University of Catalonia (UPC), Barcelona, Spain. A Bionix 358 servohydraulic mechanical testing machine (MTS Sensor Technologie GmbH & Co. KG, Lüdenscheid, Germany) with a 500 N load cell was used for applying the force and recording the bone resistance to be deformed. All tibia samples were positioned with the same orientation and the same load velocity (1 mm/min) was applied for each test.

### Statistical analysis

Statistical analysis and graphic representation were done with GraphPad Prism ver.8 (GraphPad Software). Statistical differences between groups were analyzed with a two-tailed unpaired Student’s t-test when comparing two groups, one-way analysis of variance (ANOVA), followed by Tukey as a post-hoc analysis for comparing all treatment groups, and two-way ANOVA when the gender of the animal was considered. qPCR data are expressed as relative quantity of the gene of interest versus the reference genes ± standard error of the relative quantity. All the other data are expressed as mean ± standard error of the mean (SEM). Statistical difference was accepted when p values were ≤ 0.05, and outliers were detected by Grubb’s test and removed from the analysis.

### Ethical approval

All experimental procedures involving animals were designed and performed following standard guidelines of the European Communities Council Directive 86/609/EEC and the ARRIVE guidelines. These were approved by the Institutional Animal Care and Use Committee of the Universitat Autònoma de Barcelona (M0348-DO5, approved procedure code, P1-4882) and biosecurity procedure HR-265-16.

## Data Availability

Data that support the findings of this study are available from the corresponding author upon reasonable request.
